# Ocular toxicity of psychotropic medications in a tertiary hospital in Lagos, Nigeria

**DOI:** 10.22336/rjo.2024.20

**Published:** 2024

**Authors:** Modupe Medina Balogun, Olurotimi Ayodele Coker

**Affiliations:** * Department of Surgery, Ophthalmology Unit, Lagos State University College of Medicine, Lagos State, Nigeria; ** Department of Behavioural Medicine, Lagos State University College of Medicine, Lagos State, Nigeria; *** Lagos State University Teaching Hospital, Lagos State, Nigeria

**Keywords:** toxicity, ocular, antipsychotics

## Abstract

**Objective:** This study aimed to determine the ocular toxicity of the psychotropic drugs used by patients and to proffer suggestions on how to prevent visual impairment or blindness in patients on antipsychotics.

**Methodology:** This was a prospective, hospital-based cross-sectional study. Participants were adult patients between 18 and 70 years, diagnosed with psychosis, and who had been on antipsychotic medications for at least one year. All the recruited participants had an examination of the anterior and posterior segments of the eyes done. Schirmer’s test, Tear film Break-up time (TBUT), Central Corneal thickness (CCT), Colour vision test, and Contrast sensitivity test were done. The collected data was analyzed using IBM SPSS 28.0.

**Results:** The study enrolled patients who were mainly females (55.1%). The highest age group of the cases was 29-38 years (29.7%). The examination of the eyes and investigations revealed that 10.2% of the respondents on antipsychotics had color vision deficiency and 25.4% - loss of contrast sensitivity. Lid pigmentation was observed in 20.3% and cataract in 32.2%. Degeneration of the peripheral retina was observed in 4.2% of patients on antipsychotic medication.

Schirmer’s test showed mild, moderate, and severe dry eyes in 11%,17.8%, and 20.3% of the participants respectively. The test for Central Corneal Thickness (CCT) showed 50.0% of the respondents had a thin cornea and 24.6% had a thick cornea. 17.8% of the surveyed respondents manifested high eye pressure.

Discussion: Psychotropics are the gold standard for the treatment of psychotic episodes and disorders. The choice of drug, dosing, and mode of administration depends on the severity of the psychotic disorder. Higher doses of psychotropics were reported to cause toxicity in different organs in the body including the eyes, especially on long-term use and high dosage and this can affect the quality of life of the individual negatively.

**Conclusion:** The earliest and most prominent side effect seen in patients on psychotic medication was dry eyes. There were a few cases of blinding eye diseases like glaucoma, and cataract. For these reasons, ophthalmic assessments should be included as part of the management of psychiatric patients early at the start of antipsychotic treatment.

**Abbreviations:** FGA = First Generation Antipsychotics, SGA = Second Generation Antipsychotics, TCAs = Tricyclic Antidepressants, CCT = Central Corneal Thickness, IOP = Intraocular Pressure, TBUT =Tear film Break-up Time, BIO = Binocular Indirect Ophthalmoscope

## Introduction

Mental disorders are defined by the Diagnostic and Statistical Manual of Mental Disorders-5 as a syndrome characterized by clinically significant disturbance in an individual’s cognitive, emotional regulation or behavior that reflects a dysfunction in the psychological, biological, or developmental process underlying mental functioning [**1**], in which psychosis is an intrinsic component. Psychosis can be distressing to the patient with a sense of poor quality of life, and this could constitute a burden to the caregiver and a financial burden on the nation.

The mainstay for psychotic treatment is antipsychotic medications that provide relief from psychotic symptoms for many patients [**2**] and psychotherapy with social support. Treatment might be long-term in some cases. Psychotropic drugs are divided into first-generation antipsychotics (FGA) or typical antipsychotics that work by inhibiting dopaminergic neurotransmission. They also have noradrenergic, cholinergic, and histaminergic blocking actions [**3**]. Second-generation antipsychotics(SGA) or atypical antipsychotics work by blocking D2 dopamine receptors and serotonin receptor antagonist action [**3**]. Drugs taken orally are systematically absorbed with the potential to cause drug reactions in all parts of the body including the eyes [**4**]. After the liver, the eye is the second most frequent organ to manifest drug toxicity. Despite its small mass, it has a rich blood supply and the visual system consists of numerous tissues derived from different origins [**5**]. The eye also exhibits a very high metabolic rate. All these factors are important for the human eye to be sensitive to psychotropic drugs.

Ocular toxic effects due to antipsychotics can be divided into seven categories: eyelid and kerato-conjunctival disorders, uveal tract disorders, accommodation interference, angle-closure glaucoma, cataract and pigmentary deposits on the lens and cornea, retinopathy and other disorders [**6**]. These toxicities are dose and duration-related [**7**] and some can be sight-threatening, while others may lead to blurred vision and discomfort.

The disorders of the eyelid and keratoconjunctiva are related to Phenothiazine and Lithium. At the same time, high levels of Chlorpromazine can cause abnormal pigmentation of the eyelids, inter-palpebral conjunctiva, and cornea [**8**], in which the pigment, melanin, or melanin-like substance accumulates in areas of skin and the eyes that are exposed to sunlight in some patients on chronic Chlorpromazine therapy. The dose and duration of exposure to the drug are important in the development of abnormal skin pigmentation [**9**] though the pigmentation is said to be completely reversible on stoppage of the drug. In a case report on a psychiatric patient on Chlorpromazine for seven years, blue-gray pigmentations were observed on the skin, including the cornea and the lens, which disappeared after 10 months of discontinuation of the drug, except for that on the cornea [**10**].

Tricyclic antidepressants (TCAs) were reported to induce accommodation interference in the form of mydriasis and cycloplegia due to the anticholinergic effect of these medications. This was evident in drugs like Risperidone, which can cause transient episodes of visual disturbances that disappear on reduction of the drug to a low daily dose [**11**].

Glaucoma is defined as a group of diseases that is characterized by optic neuropathy and visual field defects, in which increased intraocular pressure (IOP) is a major risk factor. Angle-closure secondary to psychotropic medications is a rare but visually threatening side effect. The mechanism by which this occurs is due to their anticholinergic effect resulting in mydriasis and forward displacement of the lens-iris diaphragm and ciliary body swelling or serotonergic effect, which results in the lens-iris diaphragm displacement and relaxation of sphincter pupillae muscle [**12**]. These changes can cause occlusion of the trabecular meshwork, blocking the drainage of aqueous humor with an increase in IOP in susceptible individuals. The highest risk classes are tricyclic antidepressants (TCAs) and second-generation antipsychotics (SGAs), evident in the documented case reports [**12**-**14**].

Similarly, lens changes have been reported with the use of antipsychotic drugs. High doses of typical antipsychotics like Chlorpromazine for the long term had been implicated in the formation of cataract. Doses as high as 800 mg/day for 2 years can produce pigmentary deposits on the lens [**15**]. The opacity of the lens can also occur, though rare on prolonged use of atypical antipsychotics such as Clozapine [**16**]. The mechanisms involved in the formation of these opacities are through photosensitizing agents like Chlorpromazine and its metabolites denature proteins, which become opacified when exposed to sunlight and are deposited in the skin, lens, and cornea [**15**]. In the same vein, endogenous melanin traps free radicals produced by certain psychotropic agents like Chlorpromazine, and the resulting compound shows lens discoloration [**8**]. This phototoxic reaction creates cataract cellular changes.

The retina, an extension of the brain, has functions mediated by neurotransmitters and ion movements that are directly or indirectly affected by psychotropic agents [**8**]. The most common lesion documented is pigmentary retinopathy. Retinopathy is related to high doses of typical antipsychotics, mainly Chlorpromazine and Thioridazine. Pigments are gradually deposited from the periphery to the central retina producing loss of peripheral vision, decreased night vision, central scotoma, and total blindness [**8**]. Most cases are associated with Thioridazine use than with Chlorpromazine and follow large dosages and long-term use. The effect appears much earlier within weeks [**8**].

As some of these side effects can lead to severe visual impairment or irreversible blindness, there is a paucity of information found in the literature on studies in low and middle-income countries, especially in sub-Saharan countries, on these toxicities. Thus, this study aimed to determine the ocular toxicity of the psychotropic drugs used by patients of the Department of Psychiatry of the Lagos State University Teaching Hospital (LASUTH), the statistical associations between psychotropic medications and ophthalmic disorders and to proffer suggestions on how to prevent visual impairment or blindness in patients receiving treatment for chronic mental disorders with psychosis.

## Methodology

This was a prospective, hospital-based cross-sectional study that took place in the Departments of Psychiatry and Ophthalmology of Lagos State University Teaching Hospital, Lagos, Nigeria.

Participants were consecutive adult patients between 18 and 70 years diagnosed with psychosis and who had been on antipsychotic medications for at least one year and were recruited for the study from January to June 2023.

All the recruited participants underwent an examination of the anterior segment of the eyes with a slit-lamp and posterior segment examination with the Binocular Indirect Ophthalmoscope (BIO) and a visual acuity check with the Snellen’s chart. Schirmer’s test without anesthesia [**17**] to measure both basic and reflex tears and Tear film Break-up time (TBUT) [17].

Central Corneal thickness (CCT) was measured using the ultrasound pachymeter. The color vision test was done with the Ishihara plates, while the Contrast sensitivity test was done using the Pelli Robson contrast sensitivity chart.

The collected data was analyzed using IBM SPSS 28.0. Descriptive statistics were used to determine the frequency and percentage distribution of variables. Cross-tabulation was used to analyze the association between categorical variables, and a chi-square test was used to assess the significance of this association. The significance level (p-value) was set at < 0.05.

## Results

**Table 1** shows the demographic and other characteristics of the participants. Regarding the gender of the patients, there were mainly females (55.1%). The highest age group of the cases was between 29-38 years (29.7%), while the 69-78 age range had the lowest representation of cases accounting for 3.4%. Most of the cases were single (47.5%), and a significant number of them went to higher institutions (54.2%). A high proportion of patients (55.1%) had been on antipsychotic medications within 1-5 years, 24.6% on drugs for 5-10 years, and 20.3% had been using drugs for more than ten years. Most of those on medications were on single-drug therapy (65.3%), mainly Olanzapine tablets (41.5%) and Risperidone (9.5%), while 29.7% were on two-drug combinations, mainly Fluphenazine and Olazepine (12.7%), and 5.0% on a multi-drug combination, mainly Olanzipine, Amitriptyline, and Fluphenazine.

**Table 1:** Sociodemographic and other characteristics of the participants

Variable	Frequency (n=118)
Male	53 (44.9%)
Female	65 (55.1%)
Age (years)	
18-28	7 (14.4%)
29-38	35 (29.7%)
39-48	30 (25.4%)
49-58	20 (16.9%)
59-68	12 (10.2%)
69-78	4 (3.4%)
Educational level	
Informal	0 (0.0%)
Primary	14 (11.9%)
Secondary	40 (33.9%)
Higher Institution	64 (54.2%)
Marital Status	
Single	56 (47.5%)
Married	48 (40.7%)
Divorced	5 (4.2%)
Separated	9 (7.6%)

Examination of the eyes and investigations revealed that 10.2% of the respondents on antipsychotics had color vision deficiency and the difference was statistically significant (p.0.00). Moderate loss of contrast sensitivity was observed in 25.4% of the cases and severe loss in 11.9% and the difference was statistically significant. Anterior segment examination showed lid pigmentation in 20.3% of the cases, conjunctiva pigmentation in 16.1%, lens pigmentation in 3.4%, corneal endothelial lysis in 1.7%, pupillary mydriasis in 2.5%, and cataract in 32.2%. Examination of the posterior segment revealed degeneration of the peripheral retina in 4.2% of the patients on antipsychotic medications and pale disc in 16.1% of them. Abnormal extra-ocular muscle movement was seen in 5.1% of the cases as shown in **Table 2**.

**Table 2:** Ocular complications and diagnosis of the participants

Variable	Case (%) n=118	X2 (P-value)
Color Vision (CV) Test		
CV Deficiency	12 (10.2)	8.5 (0.00)*
Normal CV	106 (89.8)	
Contrast Sensitivity (CS) Test		
Normal CS	74 (62.7)	22.1 (0.00)*
Moderate loss	30 (25.4)	
Severe loss	14 (11.9)	
Lid Pigmentation		
Absent	94 (79.7)	23.6 (0.00)*
Pigmented	24 (20.3)	
Conjunctiva Pigmentation		
Absent	99 (83.9)	15.1 (0.00)*
Pigmented	19 (16.1)	
Lens pigmentation		
Absent	114 (96.6)	0.6 (0.40)
Pigmented	4 (3.4)	
Cornea		
Normal	116 (98.3)	2.0 (0.15)
Endothelial Lysis	2 (1.7)	
Anterior chamber depth		
Normal	114 (96.9)	1.8 (0.38)
Deep	4 (3.4)	
Shallow	0 (0.0)	
Pupil		
Reactive	115 (97.5)	0.1 (0.70)
Dilated (Mydriasis)	3 (2.5)	
Lens		
Clear	80 (67.8)	0.1 (0.78)
Cataract	38 (32.2)	
Retina		
Normal	113 (95.8)	5.1 (0.02)
Degeneration of periphery	5 (4.2)	
Optic Nerve		
Normal disc	99 (83.9)	2.1 (0.14)
Pale disc	19 (16.1)	
Extraocular Muscle Movements (EOMM)		
Normal	112 (94.9)	2.0 (0.15)
Abnormal	6 (5.1)	
*Statistically significant		

Concerning visual acuity, 14.4% of the participants had mild visual impairment, 16.9% had moderate, and 3.4% had severe visual impairment. Regarding Schirmer’s test, mild, moderate, and severe dry eyes were observed in 11%,17.8%, and 20.3% of the participants, respectively. The test for Central Corneal Thickness (CCT) showed that 50.0% of the respondents had a thin cornea, and 24.6% had a thick cornea. 17.8% of the surveyed respondents manifested high eye pressure, as shown in **Table 3**. 

**Table 3:** Ocular complication assessments of the participants

Variable	Case (%) n=118	X2 (P-value)
Schirmer’s test (mm)		
Severe dry eyes (0-5)	24 (20.3)	3.813 (0.282)
Moderate dry eyes (5-10)	21 (17.8)	
Mild dry eyes (10-15)	13 (11.0)	
Normal tear function (> 15)	60 (50.8)	
TBUT test (S)		
Dry eyes (< 10)	60 (50.8)	0.271 (0.602)
Normal eyes (>10)	58 (49.2)	
Visual Acuity		
Normal (6/4-6/9)	77 (65.3)	5.854 (0.119)
Mild (6/12-6/18)	17 (14.4)	
Moderate (6/18-6/60)	20 (16.9)	
Severe (6/60-3/60)	4 (3.4)	
Central Cornea Thickness (µm)		
Thin cornea (< 540)	59 (50.0)	2.159 (0.340)
Normal cornea (540-560)	30 (25.4)	
Thick cornea (> 560)	29 (24.6)	
Intraocular Pressure		
Normal	97 (82.2)	0.00 (1.00)
High pressure	21 (17.8)	

**Table 4** shows the association between the duration of antipsychotic medications and the different ocular problems. Assessment showed that with Schirmer’s test, the patients on antipsychotics for more than ten years had a higher proportion of mild to moderate dry eyes, accounting for 20.8% of the cases, whereas, those on medications for 5 years or less had the highest frequency of severe dry eyes, with a prevalence rate of 23.1%. TBUT revealed a significant proportion (56.9%) of individuals on medications for 1-5 years who had dry eyes. Thin cornea was observed in 54.0% of respondents who had been on medications for over 10 years, while 43.3% of those who had been on medications for 5-10 years had thick cornea. The difference was statistically significant.

A significant proportion (25.0%) of those on medications for more than 10 years had high intraocular pressure. Moderate and severe visual impairments were observed in 20.8% and 8.3% respectively of those who had been on medications for over 10 years. 

**Table 4:** Association of medication duration with assessments of ocular complications

Variable	Duration of Medication (yrs.)			X2 (P-value)
	1-5years (%)	5-10years (%)	> 10years (%)	
Schirmer test (mm)				
Severe dry ocular (0-5)	15 (23.1)	5 (17.2)	4 (16.7)	9.034 (0.434)
Moderate dry ocular (5-10)	10 (15.4)	6 (20.7)	5 (20.8)	
Mild dry ocular (10-15)	4 (6.2)	4 (13.8)	5 (20.8)	
Normal tear function (> 15)	36 (55.4)	14 (48.3)	10 (41.7)	
TBUT test (S)				
Dry ocular (< 10)	37 (56.9)	12 (41.4)	11 (45.8)	2.513 (0.473)
Normal ocular (>10)	28 (43.1)	17 (58.6)	13 (54.2)	
Visual Acuity				
Normal (6/4-6/9)	46 (70.8)	18 (62.1)	13 (54.2)	13.09 (0.158)
Mild (6/12-6/18)	6 (9.2)	7 (24.1)	4 (16.7)	
Moderate (6/18-6/60)	11 (16.9)	4 (13.8)	5 (20.8)	
Severe (6/60-3/60)	2 (3.1)	0 (0.0)	2 (8.3)	
Central Cornea Thickness (µm)				
Thin cornea (< 540)	34 (52.3)	12 (41.4)	13 (54.2)	16.42 (0.012)*
Normal cornea (540-560)	19 (29.2)	3 (10.3)	8 (33.3)	
Thick cornea (> 560)	12 (18.5)	14 (43.3)	3 (12.5)	
Intraocular pressure (mmHg)				
Normal Eye pressure (12-21)	53 (81.5)	26 (89.7)	18 (82.2)	1.97 (0.578)
High pressure (> 21)	12 (18.5)	3 (10.3)	6 (25.0)	

**Table 5** shows the association between individual antipsychotic medications and TBUT. The findings showed a significant proportion of participants on Haloperidol (100%), Risperidone (81.8%), Chlorpromazine (71.4%), and Fluphenazine Decanoate injection (66.7%) manifested with dry eyes. On the association of Schirmer’s test and various antipsychotic medications, mild dry eyes were observed in 28.6% of those on Chlorpromazine. The association between antipsychotic medication and the Schirmer’s test did not show any significant association. However, those on Clozapine manifested higher indices of dry eyes compared to other neuroleptics, as shown in **Table 6**. 

**Table 5:** Association between antipsychotic medications and Tear Break-up Time (TBUT)

Type of Drugs	TBUT		X2 (P-value)
	Dry eyes	Normal eyes	
Olanzapine Tab	20 (40.8)	29 (59.2)	12.049 (0.061)
Clozapine Tab	0 (0.0)	2 (100.0)	
Chlorpromazine Tab	5 (71.4)	2 (28.6)	
Risperidone Tab	9 (81.8)	2 (18.2)	
Aripriprazole Tab	1 (33.7)	2 (66.7)	
Haloperidol Tab	2 (100.0)	0 (0.0)	
IM Fluephenazine Decanoate	2 (66.7)	1 (33.3)	

**Table 6:** Association between antipsychotic medications and Schirmer’s Test

Type of Drugs	Schirmer’s test				X2 (P-value)
	Severe dry eyes	Moderate dry eyes	Mild dry eyes	Normal tear function	
Olanzapine Tab	6 (12.2)	12 (24.5)	6 (12.2)	25 (51.0)	19.465 (0.364)
Clozapine Tab	2 (100.0)	0 (0.0)	0 (0.0)	0 (0.0)	
Chlorpromazine Tab	2 (28.6)	0 (0.0)	2 (28.6)	3 (42.9)	
Risperidone Tab	3 (27.3)	1 (9.1)	1 (9.1)	6 (54.5)	
Aripriprazole Tab	1 (33.3)	1 (33.3)	0 (0.0)	1 (33.3)	
Haloperidol Tab	0 (0.0)	0 (0.0)	0 (0.0)	2 (100.0)	
IM Fluephenazine Decanoate	1 (33.3)	0 (0.0)	0 (0.0)	2 (66.7)	

**Table 7** shows the association between various antipsychotic drugs and corneal thickness among the participants. The difference did not show any statistical significance. Nonetheless, those on Aripiprazole and Fluphenazine Decanoate experienced more of a thin cornea when compared to others on other antipsychotics.

The association between IOP and the different medications showed higher ocular pressure in patients on Risperidone tablets (27.3%) and Olanzapine (18.4%).

**Table 7:** Association between antipsychotic medications and corneal thickness in participants

Type of Drugs	Central cornea Thickness			X2 (P-value)
	Thin cornea	Normal Cornea	Thick cornea	
Olanzapine Tab	25 (51.0)	14 (28.6)	10 (20.4)	7.517 (0.822)
Clozapine Tab	1 (50.0)	1 (50.0)	0 (0.0)	
Chlorpromazine Tab	6 (85.7)	1 (14.3)	0 (0.0)	
Risperidone Tab	6 (54.5)	2 (18.2)	3 (27.3)	
Aripriprazole Tab	2 (66.7)	0 (0.0)	1 (33.3)	
Haloperidol Tab	1 (50.0)	1 (50.0)	0 (0.0)	
IM Fluephenazine Decanoate	2 (66.7)	1 (33.3)	0 (0.0)	

## Discussion

This study evaluated the ocular toxicity of the psychotropic drugs used by the surveyed respondents, the statistical associations between psychotropic medications and ophthalmic disorders, and tried to proffer suggestions on how to prevent visual impairment or blindness in patients receiving treatment for chronic mental disorders with psychosis.

Psychotropics are the gold standard for the treatment of psychotic episodes and disorders. The choice of drug, dosing, and mode of administration depends on the severity of the psychotic disorder. Higher doses of psychotropics were reported to cause toxicity in different organs in the body including the eyes, especially on long-term use and high dosage and this can affect the quality of life of the individual negatively [**18**]. A significant proportion of the cases in this study were on mono-drug therapy (65.3%), mainly Olanzapine tablets (41.5%), which is an atypical antipsychotic. This was similar to a study done in France, where most of the patients (73.6%) were on monotherapy and 59.4% of them on atypical antipsychotics [**19**]. This could be because atypical antipsychotics are known to be more effective than typical antipsychotics in treating negative symptoms, cognitive impairment, and mood symptoms, as well as reducing the risk for suicide and decreasing aggression [**20**] with less extrapyramidal side effects [**21**].

Color vision deficiency was not a common occurrence in this study. The difference was statistically significant. This could be due to the tool that was used to test color vision- Ishihara plates, which are not as sensitive as the Cambridge Colour test [**22**], which is a computer-based test or the Farnsworth D-15 dichotomous color blindness test that showed 71.7% of Schizophrenic patients examined have various types of color vision deficiencies [**23**].

Loss of contrast sensitivity was not a significant occurrence in this study though moderate loss was seen in 25.4% and 11.9% had severe loss. This was contrary to other studies [**24**,**25**], in which contrast sensitivity was found to be loss of vision in Schizophrenic patients on medication than in healthy individuals.

Of the 118 participants examined, visual acuity was normal in 65.3% of them, mild visual impairment in 14.4%, moderate impairment in 16.9%, and 3.4% had severe impairment. This aligned with previous studies [**26**,**27**] that showed visual acuity to be normal in 82.0% of the cases observed. In a study done in Gambia [**28**], only 1.96% of the mentally handicapped patients examined had visual impairment. However, the results were contrary to another study [**29**], in which most (67.7%) of those examined had visual impairment.

Pigmentations of the lids, conjunctiva, lens, and cornea were observed in the participants. However, the number of cases without pigmentation was statistically significant. Pigmentation was linked more to the use of Chlorpromazine and Clozapine. The ocular deposits are believed to be composed of melanin and the phototoxic drug compound. Photosensitization of the tissue proteins occurs in areas of increased sun exposure after the accumulation of the drugs in these tissues [**30**]. This was evident in a review article in Australia [**31**] that showed skin and ocular changes in the cumulative dosage of Chlorpromazine. Most of the respondents on medication in this study were on Olanzapine.

The respondents on poly-pharmacy were exposed to several drugs that can cause raised intraocular pressure, which can lead to blindness. In this study, high intraocular pressure was observed in 17.8% of the respondents. This was observed more in those who had been on antipsychotics for more than 10 years, especially on Olanzapine tablets. This finding is in line with a study [**32**] in which 11.0% of the subjects examined had raised IOP, but contrary to a study in Nigeria in which only 5.9% of those examined had glaucoma [**27**]. The low occurrence of glaucoma in this study could be a result of the low prevalence of some of the risk factors for Angle-closureGlaucoma among the cases like shallow anterior chamber and pupillary mydriasis, which was observed in 2.5% of the cases.

Antipsychotic use in the treatment of psychotic disorders has been associated with various side effects including cataract formation, which is a leading cause of reversible blindness worldwide. In this study, cataract was observed in 32.2% of the respondents. This finding was similar to a study [**33**] in which cataract was observed in 33% of the cases observed. A lower rate of cataract was observed in other studies [**26**,**27**]. The relatively low occurrence in this study could be because the cataract is an infrequent side-effect of therapy with Olanzapine, Ziprasidone, and Quetiapine with rates lower than in the general population [**33**], but it results more from high dosages of typical antipsychotics for a prolonged period [**34**].

The eye is developmentally and anatomically an extension of the brain and the retina is a continuum and, thus, can be affected by medications that have psychotropic effects. Deposition of the drugs through the vascular supply can affect the retinal pigment epithelium and the neuro-sensory retina causing pigmentary retinopathy. Pigmentary degeneration at the retina periphery was not a prominent feature in this study. Retinal toxicity is associated with Phenothiazine use, mainly Thioridazone [**35**], and, in this study, very few cases were on Phenothiazine.

Antipsychotic medications are considered risk factors for dry eyes due to their influence on the tear film status. Schirmer’s test, which indicates the quantity of tears produced, was observed to be low in 49.1% of the respondents. Participants on antipsychotic medications for more than 10 years had a higher occurrence of mild to moderate dryness of the eyes, while severe dry eyes were observed more in those on medication for 5 years or less. Severe dry eyes were observed more in patients on Clozapine (100%), moderately dry eyes in those on Aripiprazole (33.3%), and mild dry eyes in those on Chlorpromazine tabs (28.6%). The TBUT that showed stability of tears was observed to be reduced in 50.8% of the respondents, while 56.9% of those with dry eyes had been on medication for 1-5 years. These findings aligned with a study in India [**36**], in which 32% of those examined on antipsychotic therapy had dry eye disease. A study on schizophrenia patients on Clozapine and control showed dry eye disease in patients on medication [**37**]. Central corneal thickness in 50.0% of the respondents on medications had thin cornea and 24.6% had thick cornea. This was in line with a study on schizophrenia patients and control that showed thin cornea in patients on antipsychotic medications [**37**]. Central corneal thickness was also found to reduce in dry eyes [**38**]. A thin cornea will present an underestimation of the measured IOP, which may be high, thus the risk of glaucoma [**39**].

## Conclusion

The earliest and most prominent side effect observed in patients on psychotic medications was dry eyes, though it does not cause blindness, it causes discomfort, which can be devastating. There were a few cases of blinding eye diseases like glaucoma, and cataract. For these reasons, ophthalmic assessments should be included as part of the management of psychiatric patients especially at the commencement of antipsychotic treatment and if possible yearly to detect and treat complications early, thus to prevent blindness.

**Conflict of Interest Statement**

The authors state no conflict of interest.

**Informed Consent and Human and Animal Rights Statement**

Informed consent has been obtained from all individuals included in this study.

**Authorization for the use of human subjects**

Ethical approval: The research related to human use complies with all the relevant national regulations, and institutional policies, as per the tenets of the Helsinki Declaration, and has been approved by the review board Lagos State University Teaching Hospital, Lagos State, Nigeria (LREC/06/10/2011. 24/11/2022).

**Acknowledgments**

None.

**Sources of Funding**

None.

**Disclosures**

None.
